# Airway acidification impaired host defense against *Pseudomonas aeruginosa* infection by promoting type 1 interferon β response

**DOI:** 10.1080/22221751.2022.2110524

**Published:** 2022-09-14

**Authors:** Yang Liu, Ying-Zhou Xie, Yi-Han Shi, Ling Yang, Xiao-Yang Chen, Ling-Wei Wang, Jie-Ming Qu, Dong Weng, Xiao-Jian Wang, Hai-Peng Liu, Bao-Xue Ge, Jin-Fu Xu

**Affiliations:** a Department of Respiratory and Critical Care Medicine, Shanghai Pulmonary Hospital, School of Medicine, Tongji University, Shanghai, People’s Republic of China; b Institute of Respiratory Medicine, School of Medicine, Tongji University, Shanghai, People’s Republic of China; c Department of Pulmonary and Critical Care Medicine, Second Affiliated Hospitial of Fujian Medical University, Respiratory Medicine Center of Fujian Province, Fujian, People’s Republic of China; d Department of Respiratory Diseases and Critic Care Unit, Shenzhen Institute of Respiratory Disease, Shenzhen Key Laboratory of Respiratory Disease, Shenzhen People’s Hospital, Shenzhen, People’s Republic of China; e Department of Pulmonary and Critical Care Medicine, Ruijin Hospital, Shanghai Jiaotong University School of Medicine, Shanghai, People’s Republic of China; f Institute of Immunology and Bone Marrow Transplantation Center, The First Affiliated Hospital, School of Medicine, Zhejiang University, Zhejiang, People’s Republic of China; g Clinical and Translational Research Center, Shanghai Pulmonary Hospital, Tongji University School of Medicine, Shanghai, People’s Republic of China

**Keywords:** Bronchiectasis, *P. aeruginosa*, airway acidification, outer membrane vesicles, type I interferonβ

## Abstract

Airway microenvironment played an important role in the progression of chronic respiratory disease. Here we showed that standardized pondus hydrogenii (pH) of exhaled breath condensate (EBC) of bronchiectasis patients was significantly lower than that of controls and was significantly correlated with bronchiectasis severity index (BSI) scores and disease prognosis. EBC pH was lower in severe patients than that in mild and moderate patients. Besides, acidic microenvironment deteriorated *Pseudomonas aeruginosa (P. aeruginosa)* pulmonary infection in mice models. Mechanistically, acidic microenvironment increased *P. aeruginosa* outer membrane vesicles (PA_OMVs) released and boosted it induced the activation of interferon regulatory factor3 (IRF3)-interferonβ (IFN-β) signalling pathway, ultimately compromised the anti-bacteria immunity. Targeted knockout of IRF3 or type 1 interferon receptor (IFNAR1) alleviated lung damage and lethality of mice after *P. aeruginosa* infection that aggravated by acidic microenvironment. Together, these findings identified airway acidification impaired host resistance to *P. aeruginosa* infection by enhancing it induced the activation of IRF3-IFN-β signalling pathway. Standardized EBC pH may be a useful biomarker of disease severity and a potential therapeutic target for the refractory *P. aeruginosa* infection. The study also provided one more reference parameter for drug selection and new drug discovery for bronchiectasis.

## Introduction

Non-cystic fibrosis bronchiectasis was a chronic respiratory disease characterized by a clinical syndrome of cough, excessive sputum production and radiologically by an irreversible enlargement of the bronchi [1]. The prevalence of bronchiectasis had been increased globally over the past twenty years [2–4], causing a heavy economic burden to the society. *P. aeruginosa* was a conditionally pathogenic gram-negative bacterium, which was one of the most important contributor in the pathogenesis of bronchiectasis [5,6]. *P. aeruginosa* colonization was associated with an increased risk of exacerbation, more severe lung tissue destruction and more pronounced decline in lung function, but the efficacy and safety of most current exploratory therapies for *P. aeruginosa* infection were controversial [5,7,8].

Airway infection and inflammation played an important role in the development of the bronchiectasis [9,10]. The measurement of markers in EBC had the advantages of non-invasive safety, convenience, high reproducibility, multiple detection, avoiding interference with the disease itself, which had been proven to be a useful method for assessing and monitoring airway inflammation [11–13]. Currently, pH was considered to be one of the most valuable airway microenvironment indicators in EBC [14,15], and airway acidification had been reported in many inflammatory airway diseases [16–18]. However, when it came to bronchiectasis, studies were relatively scarce. As an important indicator of the airway microenvironment, there was an urgent need for high-quality prospective studies to clear the airway microenvironment pH in bronchiectasis and to clarify its relationship with disease severity and prognosis. Mechanically, whether acidic microenvironment affects *P. aeruginosa* infection remains unclear.

OMVs were produced by controlled blistering of the outer membrane of gram-negative bacteria, affecting a variety of biological processes such as factor transport, antibiotic and intercellular communication [19–21]. Lipopolysaccharide (LPS) was one of the most abundant components of OMVs, other components include proteins, DNA, RNA and metabolites [20]. *P. aeruginosa* was a classic gram-negative bacterium that release OMVs, and related components could activate NOD signals and NF-kB, which were key driver of inflammatory damage in the host [22,23]. However, whether there was other novel pathogenicity induced by OMVs remains unclear. The release of OMVs was affected by stressful conditions such as iron limitation, antibiotics and temperature, etc [24–28], while the effect of acidic microenvironment on PA_OMVs release remained unclear. We hypothesized that acidic microenvironment may affect the release of PA_OMVs and its pathogenicity. The aims of our study were to explore characteristics of airway acidification in bronchiectasis, and to clarify how it affected host defense against *P. aeruginosa* pulmonary infection and led to the deterioration of the disease.

## Materials and methods

### Clinical research

A prospective cohort study had been conducted at Shanghai Pulmonary Hospital from June 2018 to July 2020. Patients were introduced from centres of the Chinese Bronchiectasis Registry and Research Collaboration (BE-China, http://www.chinabronchiectasis.com/), and they were uniformly introduced and admitted to Shanghai Pulmonary Hospital. The protocol of this study was approved by the ethics committees of Shanghai Pulmonary Hospital (approval number K18-124) and all patients had signed the informed consent. The study was registered in the website of clinical trials (www.clinicaltrials.org; Reg. No. NCT03643263). Detailed inclusion and exclusion criteria were provided in the supplementary materials. PASS 11 software was used to calculate sample size based on the power analysis of the two-sample T-test. Previous literatures reported that the average EBC pH of patients with stable bronchiectasis was 7.2 (0.1) and that of healthy subjects was 7.7 (0.5). The proportion of patients and healthy subjects was set as 2: 1 in this study, then setting the significance level alpha (α) at 0.05 and test power 1-β at 0.9, thus, the sample size was obtained. All patients were followed up for the frequency of acute exacerbation and the time to first exacerbation during the one-year period.

### EBC collection and pH measurement

General methods for EBC collection referred to recommendations published in 2005 [29]. Detailed operation process was provided in supplementary materials.

### Bacterial culture

The *P. aeruginosa* (PAO1, ATCC-BAA-47; stain HER-1018) or GFP-PAO1 were cultured in 3 ml sterile Luria–Bertani (LB) broth for 13 h (220rpm, 37°C). Then, diluted at 1:200 and shaken for another 2 h to reach the logarithmic phase. Finally, the bacterial liquid was centrifuged at 9000rpm for 3 min, and washed with sterile PBS and suspended, then diluted with PBS to OD_600nm_ = 0.5 (equivalent of 2*10^8/mL) to be used for challenge.

### Cell culture

Thioglycollate (211716, Becton, Dickinson and Company)-elicited peritoneal macrophages from six-week female C57BL/6 mice were produced. 1*10^6 peritoneal macrophages or mouse immortalized bone marrow derived macrophages (iBMDM) were centrifuged and then seeded into 12-well culture plates and incubated overnight at 37°C in a medium of Roswell Park Memorial Institute (RPMI-1640) (GE, Hyclone, American), which supplemented with 10% fetal bovine serum (FBS, Gibco) and 1% L-glutamine-penicillin. The medium was changed to RPMI-1640 without antibiotics and culture pH of the acidic group was adjusted to 6.3 with acid before the stimulation of purified OMVs or PAO1 for different times. All reagent sources in this study were described in supplementary materials.

### Adhesion assay

Human lung adenocarcinoma cells A549 were incubated in normal or acidic (pH = 6.3) culture medium. PAO1 in logarithmic growth phases were added to the medium and incubated at 37°C for 1 h to establish bacterial adhesion. Each plate had a control well, and all of the experiments were conducted in triplicate. At the end of incubation, cells were washed with sterile PBS for at least three times and then lysed with 1 ml 1% Triton X-100 at 37°C for 10 min. The lysis suspension was diluted at different multiples and plated onto LB agar at 37°C for 18 h to confirm the bacteria load (CFU). The adhesion rate was calculated by dividing the CFU obtained after cell lysis by the total number of colonies initially added into the medium [30]. For the visualizations of adhered PAO1 to A549 cells in normal or acidic culture environments by fluorescence microscopy, cells were seeded into 15 mm glass bottom cell culture dish at 37°C in a 5% CO_2_ atmosphere and stimulated with GFP-PAO1 for 1 h to establish bacterial adhesion. After the incubation, cells were washed with PBS for three times to remove unbound bacteria, then fixed with 4% formaldehyde for 30 min at room temperature. After permeabilized with 1% Triton X-100 in PBS for 10 min, cells were stained with TRITC-phalloidin 100(nM) for 30 min at room temperature. Finally, washed cells with PBS for three times, then stained with DAPI dihydrochloride for 15 min and obtained confocal images with a Nikon confocal laser scanning microscope (60 × oil immersion objective lens).

### Bacterial intracellular survival

Mice peritoneal macrophages were incubated in RPMI-1640 medium without antibiotics at 37°C and infected with live PAO1 for 2 h. At the end of stimulation time, the supernatant was removed and replaced by RPMI-1640 medium containing gentamicin (150ug/ml) and incubated for 1 h and 3 h respectively. At the end of the incubation time, the medium was removed and the cells were washed three times with sterile PBS, then lysed with 1% Triton X-100 for 10 min. The lysis suspension was diluted at different multiples and plated onto LB agar at 37°C for 18 h to confirm the bacterial load. Percent survival of intracellular bacteria was calculated by using the following equation = (number of bacteria treated with gentamicin for 3 h/ number of bacteria treated with gentamicin for 1 h) *100%, the absolute number of intracellular survived bacteria was the number of bacteria treated with gentamicin for 1 h [31].

### Mice strains and animal experiments

IFNAR1^−/−^ mice with C57BL/6 background were bought from Cyagen Biosciences Inc (Suzhou, China). cGAS ^−/−^ mice with C57BL/6 background were provided by Prof. Ge Baoxue, Tongji University. IRF3^−/−^ mice with C57BL/6 background were provided by Prof. Wang Xiaojian, Zhejiang University. Wide type (WT) C57BL/6 mice were brought from Shanghai SLRC Laboratory Animal Center (Shanghai, China). All animals were bred in specific pathogen-free conditions at Laboratory Animal Center of Tongji University. And all animal experiments were approved by the Institutional Animal Care and Use Committee of Tongji University. Six-week-old female mice on the C57BL/6 background were used in all animal experiments, they were anaesthetized with isoflurane and were intratracheally pre-treated with lactate acid (8.0 mg/kg) or vehicle, followed by PAO1 pulmonary infection (2*10^6 CFU in 25ul PBS). The constructed airway acidification was verified in this study. Mice samples including BALF and lung tissue were collected 24 h later for further analysis. Bacterial load, IFN-β expression and production were quantified in lung tissue. Immune cell infiltrate was quantified in BALF. Neutrophils were identified as SiglecF^low/−^ CD11c^−^Ly6G^+^Ly6C^low/−^; Late apoptotic and necrotic neutrophils were gated by Apotracker™ Green+/7-ADD+ within the neutrophil population. Detailed gating strategies for flow cytometry had been added in the supplemental materials.

### Statistics

The Kolmogorov–Smirnov test was applied for analyzing the distribution of quantitative variables. Normally distributed data were presented as mean (standard deviations), non-normally distributed data were presented as median (IQR), and were compared by the independent group *t*-test or the Mann–Whitney U test respectively. Categorical variables were presented as frequencies and percentages, Chi-square test or Fisher's exact test were applied for the comparisons. Receiver operating characteristic (ROC) curve analysis was applied to verify the ability of EBC pH to predict severe bronchiectasis. Pearson correlation coefficient was used to investigate the relationship between EBC pH and some clinical parameters of patients. The Kaplan–Meier and the log-rank test were used to compare the difference of the time to first acute exacerbation in different groups. For each experiment, at least three biological replicates were performed unless otherwise indicated, log-rank testing was used to evaluate the difference in survival curves. The statistical differences in two groups were compared by using *t*-tests (two-tailed) or Mann–Whitney *U*-test. For the comparison with more than two groups, ANOVA with Bonferroni correction test was applied. All statistical analyses and diagramming were performed by the Statistical Package for Social Science (SPSS) 21.0 (Chicago, Illinois), GraphPad Prism 8.0 (San Diego, CA, USA), Origin Pro 26.0, Nano Measurer 1.2, ImageJ 1.53a and FlowJo 10.4 software. Two-sided significance level of 0.05 was chosen for all testes.

## Results

### Baseline characteristics of all subjects

**Figure 1.**
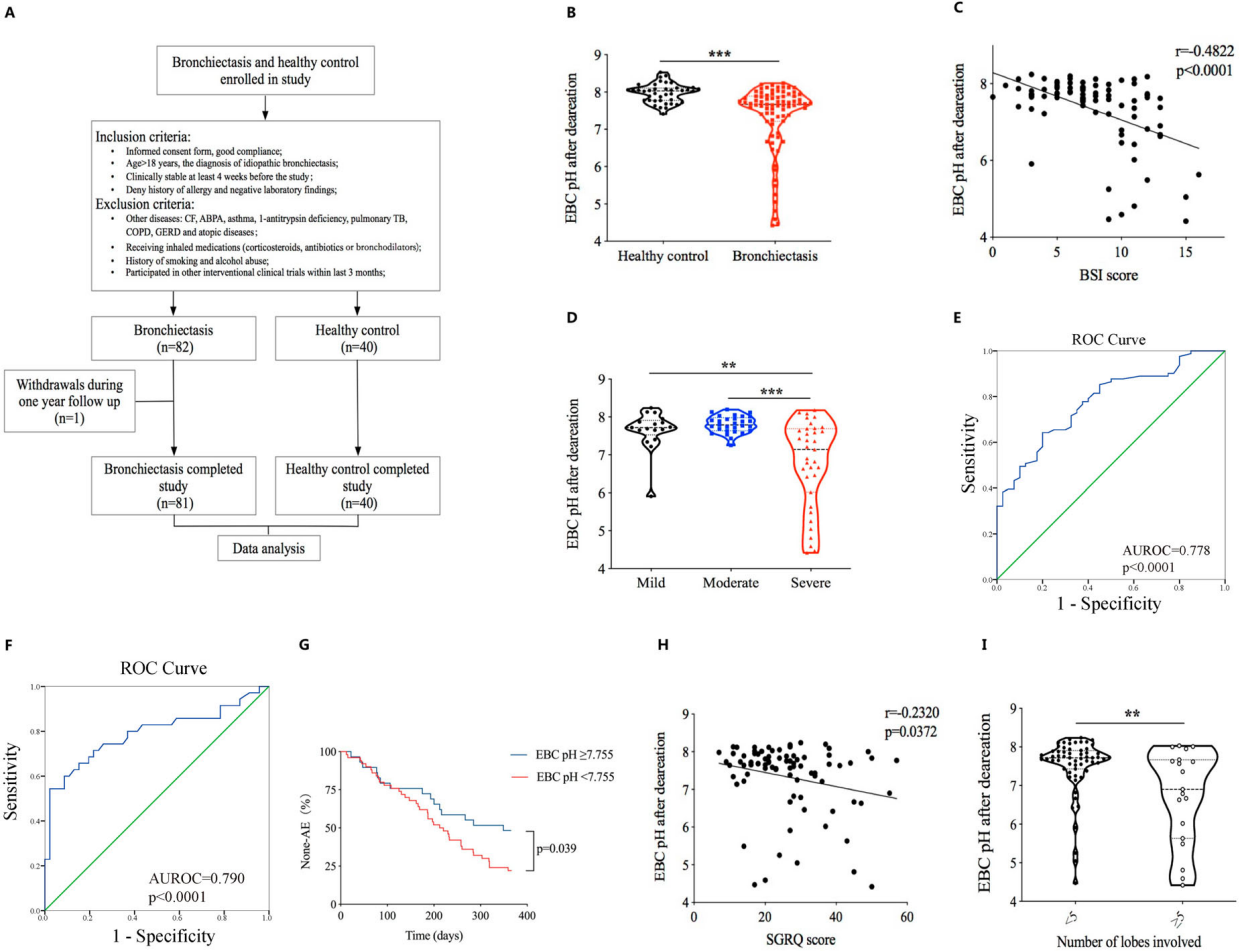
EBC pH was significantly decreased in bronchiectasis and was associated with changes of important clinical indicators. The detailed procedure of the study was shown in panel (A). Comparison of the standardized EBC pH between bronchiectasis patients and healthy control was shown in panel (B); Correlation between the standardized EBC pH and BSI score (*r* = −0.4822, *p* < 0.0001) was shown in panel (C); Comparisons of the standardized EBC pH in patients with mild, moderate and severe bronchiectasis were shown in panel (D); Receiver operating characteristic curve for the validity of the standardized EBC pH to discriminate between the health control vs bronchiectasis patients was shown in panel (E), the validity of the standardized EBC pH to discriminate between the severe bronchiectasis vs mild and moderate patients was shown in panel (F); Difference of the time to the first acute exacerbation after enrolment for patients with the standardized EBC pH less than 7.755 and those with EBC pH greater than or equal to 7.755 were shown in panel (G); Correlations between the standardized EBC pH and SGRQ score (*r* = −0.2320, *p* = 0.0372) was shown in panel (H); Comparisons of the standardized EBC pH in different subgroups such as the number of lobes involved was shown in panel (I). **p < 0.01; ***p < 0.001.

Detailed procedures of the study were shown in Figure 1(A). In total, 82 stable bronchiectasis patients and 40 healthy controls were screened in the study from 25 June 2018 to 2 July 2019 at Shanghai Pulmonary Hospital. And the data of 81 bronchiectasis and 40 controls were included in the final analysis. One patient was lost to follow up. Baseline characteristics such as age, gender distribution and BMI were similar between patients and healthy controls, additional clinical characteristics of 81 patients were shown in Table 1.

**Table 1.** Clinical characteristics and EBC pH of all subjects in both groups.

	Healthy Control (*n* = 40)	Bronchiectasis (*n* = 81)	*P* value
Age (y)	55.50 (51.00–58.75)	58.00 (47.75–64.50)	0.290
Female	25 (62.5%)	50(61.7%)	0.934
Height (m)	1.63 (1.60–1.70)	1.60 (1.57–1.68)	0.170
Weight (kg)	57.00 (50.05–63.00)	55.00 (48.00–60.00)	0.070
BMI, Kg/m^2^	21.46 (2.23)	21.03 (3.02)	0.378
BSI score	NA	7.78 (3.65)	
AE numbers in the past year	NA	1.00 (0.00–2.00)	
Duration of disease (years)	NA	15.00 (6.00–30.00)	
mMRC grading	NA	1.00 (0.00–2.00)	
SGRQ score	NA	26.52 (11.53)	
Lung lobes involved	NA	3.00 (2.00–4.75)	
Lung function	NA		
FEV1		1.58 (1.27–2.33)	
FEV1% pred		66.96 (23.58)	
FVC		2.27 (1.82–3.40)	
FVC% pred		79.62 (22.02)	
FEV1/FVC		69.07 (14.18)	
Blood gases parameters	NA		
PaO2, mmHg		86.00 (11.32)	
SaO2, %		97.20 (96.00–98.00)	
PaCO2, mmHg		40.55 (37.75–42.78)	
pH		7.41 (7.40–7.42)	
Lactic acid, mmol/L		1.25 (0.47)	
EBC pH	8.03 (7.77–8.10)	7.67 (7.22–7.89)	< 0.001

Note: Quantitative data are summarized as mean (SD) for normally distribute variables or median (IQR) for non-normally distribute variables and qualitative data are presented as n (percentage).

*p < 0.05 compared with control group.

Abbreviations: BMI: Body Mass Index; BSI: Bronchiectasis Severity Index; AE: Acute Exacerbation; SGRQ: St. George's Respiratory questionnaire; mMRC: modified British Medical Research Council; FEV1, Forced Expiratory Volume in 1 s; FVC, Forced Vital Capacity; NA, not available.

### EBC pH significantly decreased in bronchiectasis patients and was negatively correlated with the severity of the disease

Standardized EBC pH of bronchiectasis patients was significantly lower than that of healthy controls (7.67 (0.68) vs 8.03 (0.33), *p* < 0.001) (Figure 1(B)). Further analysis showed that standardized EBC pH negatively correlated with BSI scores (*r *= −0.4822, *p* < 0.001) and EBC pH of severe patients was significantly lower than that of mild and moderate patients (7.14 (1.67) vs 7.72 (0.39), *p* = 0.003; 7.14 (1.67) vs 7.79 (0.35), *p* < 0.001; respectively) (Figure 1(C,D)). Additionally, we performed an exploratory validation methodology to assess the ability of the standardized EBC pH to discriminate between health controls and patients with bronchiectasis, the average AUROC was 0.778 (95% CI 0.695–0.860, *p* < 0.001), the sensitivity was 64.2% and the specificity was 80.0%, with a cutoff EBC pH value of 7.755 (Figure 1(E)). To further assess the ability of the standardized EBC pH in distinguishing severe bronchiectasis from mild and moderate patients, the accuracy was 0.790 (95% CI 0.681–0.898, *p* < 0.001) as demonstrated by the AUROC, for a cutoff EBC pH value of 7.215, showing a sensitivity of 54.3% and specificity of 97.8% (Figure 1(F)). All subjects in this study were followed up for a year, patients with standardized EBC pH lower than 7.755 had a significantly shorter mean time to the first acute exacerbation after enrolment when compared with those with EBC pH higher than or equal to 7.755 (217.000 (95%CI 185.270–248.730) vs 257.379 (95%CI 211.897–s302.862), *p* = 0.039) (Figure 1(G)). Pearson correlation analysis showed that standardized EBC pH negatively correlated with St. George breathing questionnaire score (SGRQ) (*r* = −0.2320, *p* = 0.037) (Figure 1(H)). Moreover, further analysis revealed that patients with lung lesions involving more than or equal to 5 lobes had significantly lower standardized EBC pH than that of patients with lung lesions less than 5 lobes (6.90 (2.04) vs 7.72 (0.48), *p* = 0.007) (Figure 1(I)).

### Acidic microenvironment deteriorated *P. aeruginosa* infection in vitro and aggravated its induced type 1 interferonβ response

**Figure 2.**
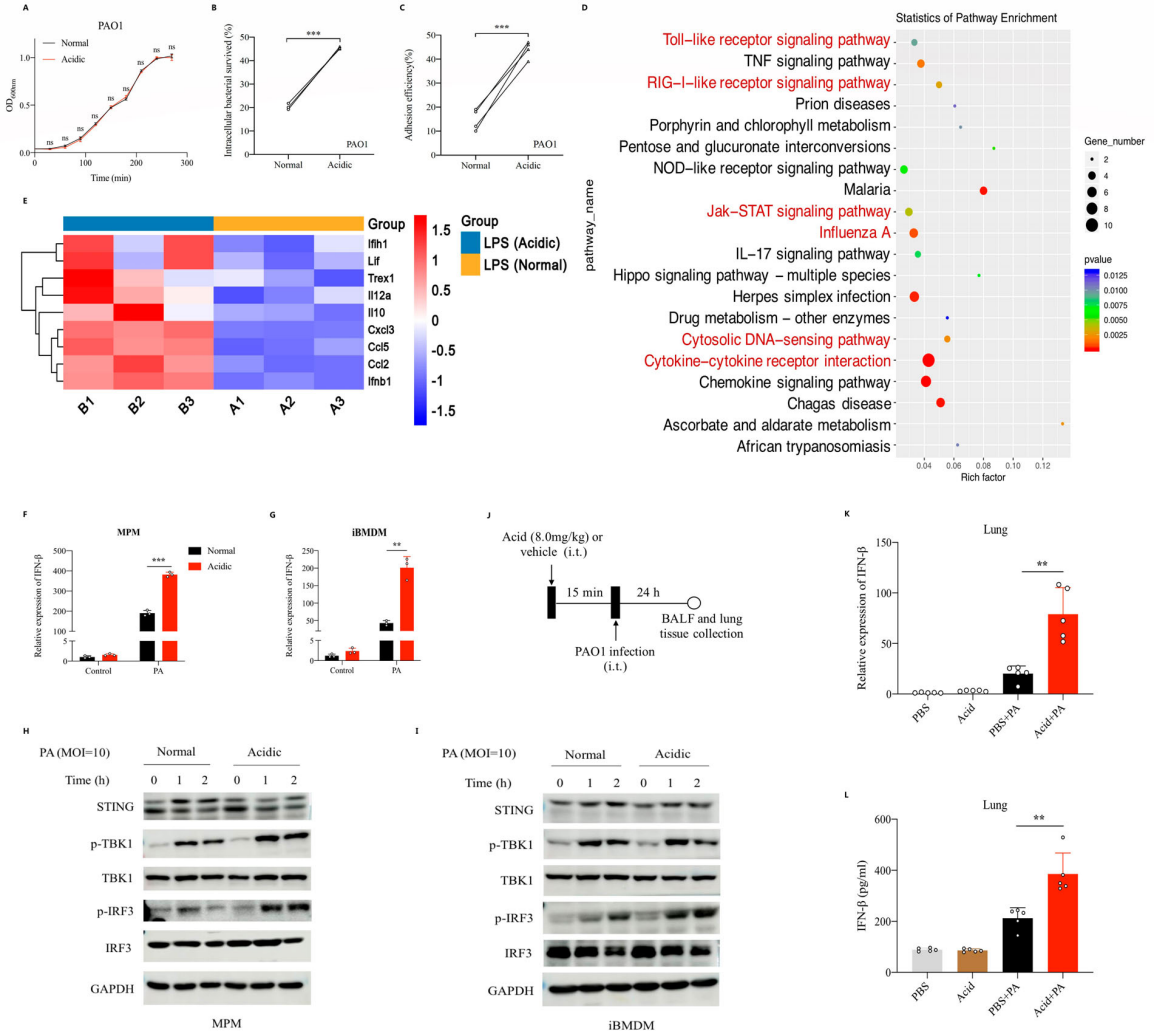
Acidic microenvironment deteriorated *P. aeruginosa* infection in vitro and aggravated its induced type 1 interferonβ response. (A) Shown was the growth curves of PAO1 in normal or acidic (pH = 6.3) bacterial culture environments; (B) Comparison of the intracellular bacterial survived rate of PAO1 in mice peritoneal macrophages in normal or acidic (pH = 6.3) cell cultures, the peritoneal macrophages were stimulated with PAO1(MOI = 0.1) for 2 h before lysis; (C) Comparison of the adhesion efficiency of PAO1 to A549 cells in normal or acidic (pH = 6.3) cell cultures, the A549 cells were stimulated with PAO1(MOI = 10) for 1 h before lysis; (D) and (E) KEGG enrichment scatterplot and the heat map showed the alteration of important genes expression in mice peritoneal macrophages following by *P. aeruginosa* LPS (1ug/ml) stimulation for 2 h in normal or acidic (pH = 6.3) cell cultures; (F) and (G) Comparisons of the IFN-β expression following by PAO1 infection in normal or acidic (pH = 6.3) cell cultures, the peritoneal macrophage and immortalized bone marrow-derived macrophages (iBMDM) of mice were stimulated with PAO1(MOI = 2) for 2 h; (H) and (I) Western blot analysis of the proteins in innate immune signalling pathway, which were isolated from peritoneal macrophage and iBMDM of mice. The peritoneal macrophage or iBMDM were stimulated with PAO1(MOI = 10) in normal or acidic (pH = 6.3) cell cultures for different time before lysis; (J) Experiment and analysis scheme for PAO1 infection after acid pretreatment. Six week old female C57BL/6 mice were intratracheally infected with PAO1(2*10^6 cfu in 25 ul PBS, per mouse) or PBS for 24 h with or without lactic acid pretreatment (8.0 mg/kg), and monitored for (K) the IFN-β gene expression and (L) IFN-β protein production in mice lung tissue; **p < 0.01; ***p < 0.001.

We explored the effect of culture environments in the growth of *P. aeruginosa* in vitro, and found that acidic microenvironment did not affect the growth of PAO1 (Figure 2(A)). To determine whether acidic microenvironment will affect the pathogenicity of PAO1, we adjusted the pH of cell cultures to 6.3 by adding acidic substances such as lactic acid. Results showed that the intracellular bacterial survival rate in mice peritoneal macrophage and the adhesion efficiency of PAO1 to A549 cells in acidic culture (pH = 6.3) were higher than that in normal condition (45.46% vs 20.37%, *p* < 0.001, 44.00% vs 14.75%, *p* < 0.001) (Figure 2(B,C), Figure S1A). Then, we stimulated mice peritoneal macrophages with *P. aeruginosa* LPS under normal and acidic (pH = 6.3) conditions respectively. The extracted cellular RNA was collected for transcriptomic analysis and results showed that acidic microenvironment could promote the activation of LPS-induced type I interferons production signalling pathway (Figure 2(D), Figure S1B-1C), and the expression of IFN-β in macrophages after LPS stimulation was significantly upregulated under acidic conditions compared with that of normal conditions (Figure 2(E)). To verify the above results, we stimulated mice peritoneal macrophages and iBMDM cells with PAO1 in normal and acidic cultures respectively, and found that acidic environment could significantly promote the expression of IFN-β following by *P. aeruginosa* stimulation (Figure 2(F,G)). We examined the activation of key proteins on the signalling pathway that induced IFN-β expression, and found that acidic environment could significantly enhance IRF3 phosphorylation followed by *P. aeruginosa* infection (Figure 2(H,I)). Besides, we established mice model and found that the mRNA expression and protein production of IFN-β induced by *P. aeruginosa* pulmonary infection were increased significantly in the acid pretreatment group compared with that in the non-pretreatment group (Figure 2(J–L)).

### Acidic microenvironment exacerbated *P. aeruginosa* pulmonary infection by aggravating type 1 interferonβ response

**Figure 3.**
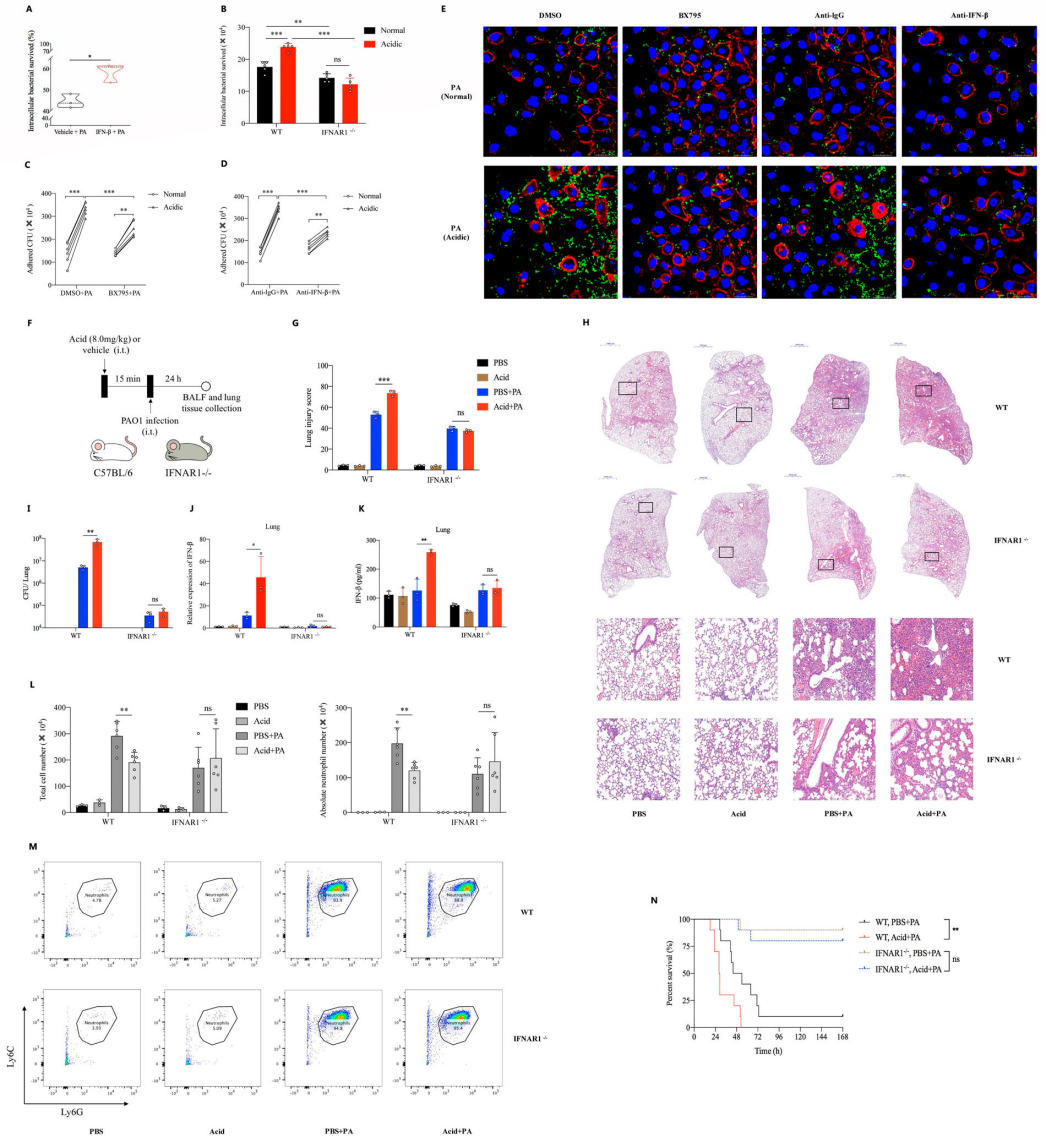
Acidic microenvironment exacerbated *P. aeruginosa* lung infection by aggravating type 1 interferonβ response in vitro and in vivo. (A) The intracellular bacterial survived rate of PAO1 in mice peritoneal macrophages that pretreated with vehicle control or 10 ng/ml recombinant human IFN-β for 1 h, then stimulated with PAO1(MOI = 0.1) for 2 h before lysis; (B) Comparisons of the intracellular survived bacteria number in peritoneal macrophages of WT and IFNAR1^−/−^ mice, cells were stimulated with PAO1(MOI = 1) for 2 h in normal or acidic (pH = 6.3) cell cultures before lysis; (C) and (D) The amounts of PAO1 adhered to A549 cells in normal or acidic (pH = 6.3) cell cultures, cells were pretreated with DMSO/IRF3 inhibitor BX795(1uM) or lgG isotype ctrl/purified anti-mouse IFN-β antibody (10ug/ml) for 1 h, then stimulated with PAO1(MOI = 10) for 1 h before lysis; (E) Visualizations of adhered PAO1 to A549 cells in normal or acidic (pH = 6.3) cell cultures by fluorescence microscopy. The A549 cells were pretreated with DMSO/IRF3 inhibitor BX795(1uM) or lgG isotype ctrl/purified anti-mouse IFN-β antibody (10ug/ml) for 1 h, then stimulated with GFP- PAO1(MOI = 10) (green) for another 1 h. Cell membrane was visualized by TRITC-phalloidin (red). Nucleus were stained with DAPI (blue); (F) Experiment and analysis scheme for PAO1 infection in WT and IFNAR1^−/−^ mice after acid pretreatment. IFNAR1 knockout mice with C57BL/6 background and six week age-matched WT mice were intratracheally infected with PAO1 (2*10^6 cfu in 25ul PBS, per mouse) or PBS for 24 h with or without lactic acid pretreatment (8.0 mg/kg), and monitored for (G) and (H) the infiltration of inflammatory cells in lung following by H&E staining and the lung injury scores caused by the infection, (I) the bacterial load in lung tissue, (J) and (K) IFN-β gene expression and protein production of mice lung tissue, (L) and (M) the immune cells classification and quantification identified in the balf of mice by flow cytometry and (N) the survival (*n* = 10 per group). *p < 0.05; **p < 0.01; ***p < 0.001.

Mice peritoneal macrophages were pretreated with pure IFN-β protein and then stimulated with PAO1, results showed that the intracellular survival rate of *P. aeruginosa* in IFN-β pretreated group was significantly higher than that of the none pretreatment group (Figure 3(A)). To further clarify whether acidic environment exacerbates *P. aeruginosa* infection by promoting the production of IFN-β, BX795 (IRF3 inhibitor) and IFNAR1^−/−^ peritoneal macrophages were used in this study. Results showed that IFN-β inhibition or blocking alleviated the increased intracellular survival of *P. aeruginosa* (Figure 3(B), Figure S2A) and inhibited the increased adhesion ability of *P. aeruginosa* to epithelial cells (Figure 3(C–E)) that promoted by acidic environment. Moreover, IFNAR1 ^−/−^ mice with C57BL/6 background were used in this study (Figure S2B). The *P. aeruginosa* pulmonary infection models with or without intratracheally lactate acid pretreatment were established in WT and IFNAR1^−/−^ mice respectively (Figure 3(F)). We found that there were significantly increased pathological damage of lung tissues (Figure 3(G,H), Figure S2C), bacterial loads (Figure 3(I)), IFN-β production (Figure 3(J,K)), mortality (Figure 3(N)) and decreased neutrophils (Figure 3(L,M)) caused by *P. aeruginosa* lung infection under acidic airway environment than those under normal condition in WT mice. However, differences in the above indicators of the same experiments in IFNAR1 ^−/−^ mice were not observed (Figure 3(G–N), Figure S2C). We further explored underlying reasons of the decreased neutrophils, results showed that there were increased neutrophil chemokines expression (Figure S2D) and late apoptotic and necrotic neutrophils (Figure S2E) induced by *P. aeruginosa* lung infection under acidic airway environment.

### PA_OMVs were actively taken up by macrophages through endocytic pathways and activated innate immune receptors to induce type 1 interferonβ production

**Figure 4.**
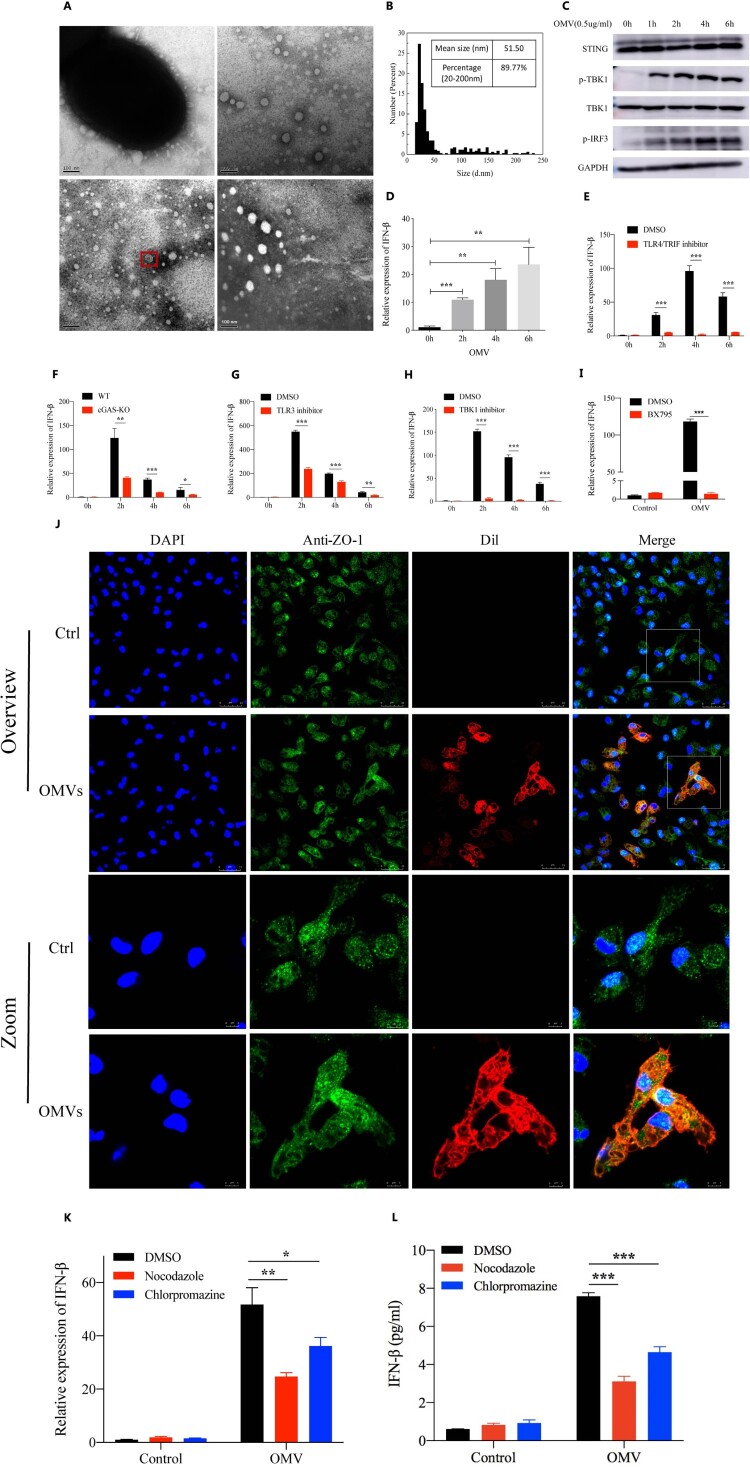
PA_OMVs were actively taken up by macrophages through endocytic pathways and activated multiple receptors to induce type 1 interferonβ production. (A) The morphology of PAO1-derived OMVs were shown by transmission electron microscope; (B) Size distribution of OMVs were determined by nano measure 1.2; (C) Western blot analysis of the proteins in innate immune signalling pathway, peritoneal macrophages of mice were stimulated with purified OMVs (0.5 ug/ml) for different time points before lysis; (D) The expression of IFN-β in peritoneal macrophages of mice following by purified OMVs (0.5 ug/ml) stimulation for different time points; (E) The expression of IFN-β following by purified OMVs (0.5 ug/ml) stimulation for different time points in peritoneal macrophages pretreated with DMSO or resatorvid (1uM) for 1 h; (F) The expression of IFN-β following by purified OMVs (0.5 ug/ml) stimulation for different time points in peritoneal macrophages of WT or cGAS KO mice; (G) and (H) The expression of IFN-β following by purified OMVs (0.5 ug/ml) stimulation for different time points in peritoneal macrophages pretreated with bafilomycin A1(1uM) or GSK8612 (1 uM) for 1 h; (I) The expression of IFN-β following by purified OMVs (0.5ug/ml) stimulation for 2 h in peritoneal macrophages pretreated with DMSO or BX795 (1uM) for 1 h; (J) Visualization of internalized OMVs by fluorescence microscopy. Peritoneal macrophages were incubated with Dil-labeled OMVs(0.5ug/ml) for 2 h at 37°C. Cell membrane was visualized by immunostaining with antibodies against the zonula occludens ZO-1 protein followed by Alexa Fluor 488-conjugated secondary antibody (green). Nucleus were stained with DAPI (blue). Internalized Dil-labeled OMVs are visualized in red; (K) and (L) IFN-β gene expression and protein production following by purified OMVs (0.5ug/ml) stimulation for 2 h in peritoneal macrophages pretreated with Nocodazole (25ug/ml) or Chlorpromazine (5ug/ml) for 1 h; *p < 0.05; **p < 0.01; ***p < 0.001.

All OMVs were successfully purified from PAO1 and verified to be devoid of bacterial contamination by agar plating (data not shown). The transmission electron microscopy images showed that PA_OMVs had the characteristic cup-shape morphology of vesicles (Figure 4(A)). Mean size distribution of PA_OMVs was 51.50 nm (95%CI 46.31–56.68 nm) and the percentage of vesicles between 20 and 200 nm was 89.77% (Figure 4(B)). To verify whether OMVs played an important role in the process of IFN-β production induced by *P. aeruginosa* infection, we stimulated the mice peritoneal macrophages with purified OMVs to collect cellular RNA and protein at different time points. Results showed that purified OMVs could activate TANK-binding kinase 1(TBK1)-IRF3 signalling pathway, leading to the phosphorylation of TBK1 and IRF3, and the expression of IFN-β (Figure 4(C,D)). Considering that contents of OMVs including LPS, DNA and RNA had now been described as strong inducers of type 1 interferons by activating different pattern recognition receptors (PRRs). In order to confirm the pathway through which PA_OMVs induced IFN-β production, different pathway inhibitors or peritoneal macrophages from cGAS -/- mice were used in this study. Results showed that PRRs, including TLR4, cyclic GMP-AMP synthase (cGAS), and TLR3 all played a role in the induction of IFN-β followed by PA_OMVs stimulation (Figure 4(E–G)). In addition, TBK1 and IRF3 were the common downstream pathway proteins of these PRRs activated by PA_OMVs (Figure 4(H,I)). Given that endocytosis was currently recognized as the main pathway for biological macromolecules uptake and these OMVs displayed a characteristic bilayer lipid structure, we next asked whether PA_OMVs were internalized by endocytosis and then activated PRRs to induce type 1 interferon signalling. In this case, we tried to intuitively show whether PA_OMVs could be taken up by macrophages. PA_OMVs were stained with the red fluorescent lipophilic compound Dil and the cell were labelled with anti-ZO1 tight junction protein antibody and DAPI. After co-incubation of macrophages with Dil-labeled PA_OMVs for 2 h, OMVs were observed in the cytoplasm of cells under confocal fluorescence microscopy, indicating that these OMVs could be internalized by macrophages (Figure 4(J)). Then, we pretreated macrophages with inhibitors of microtubule assembly and clathrin-mediated endocytosis, nocodazole and chlorpromazine, and then stimulated them with PA_OMVs. Results showed that pretreatment with nocodazole or chlorpromazine significantly reduced the expression of IFN-β mRNA as well as protein production in response to PA_OMVs (Figure 4(K,L)).

### Acidic microenvironment increased IRF3-dependent IFN-β expression by enhancing OMVs-induced IRF3 phosphorylation

**Figure 5.**
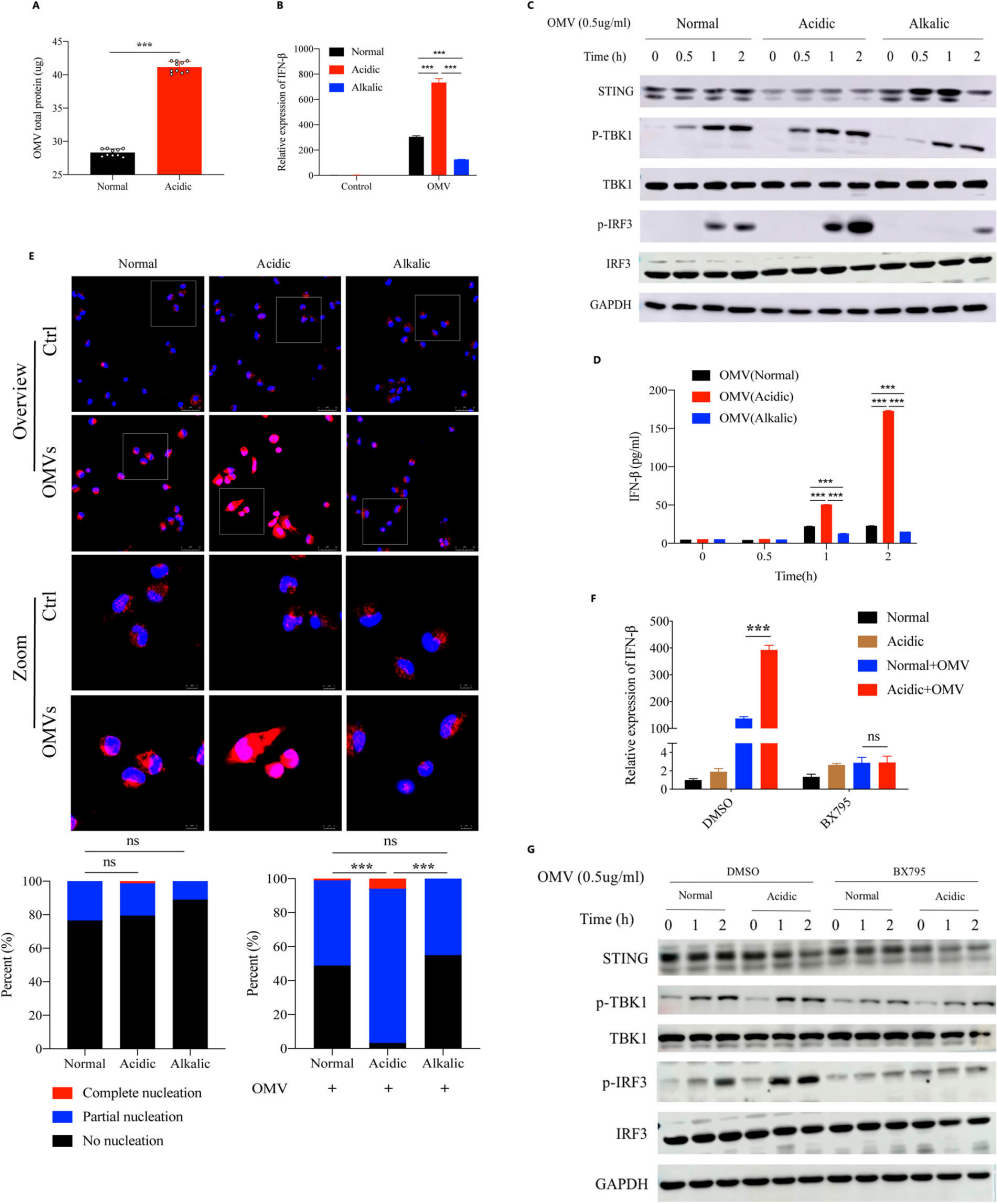
Acidic microenvironment increased IFN-β production by enhancing IRF3 activation signal induced by OMVs stimulation. (A) BCA protein quantitative analysis of OMVs total protein released by PAO1 in normal or acidic (pH = 6.3) bacterial cultures that adjusted by lactic acid; (B) The IFN-β expression following by OMVs (1.0ug/ml) stimulation for 2 h in normal, acidic (pH = 6.3) or alkalic (pH = 8.7) cell cultures; (C) Western blot analysis of the proteins in innate immune signalling pathway, peritoneal macrophages of mice were stimulated with purified OMVs (0.5 ug/ml) for different time points under different culture environments before lysis; (D) The level of IFN-β protein following by OMVs (1.0 ug/ml) stimulation for different time points under normal, acidic (pH = 6.3) or alkalic (pH = 8.7) culture environments; (E) Immunofluorescence analysis of IRF3 translocation and activation in peritoneal macrophages after OMVs (0.5 ug/ml) stimulation for 2 h in normal, acidic (pH = 6.3) or alkalic (pH = 8.7) culture environments. The nuclear translocation and activation of IRF3 was calculated by calculating the proportion of IRF3 that enters the nucleus completely, partially enters the nucleus, and does not enter the nucleus at all. At least 10 microscopic fields with more than 300 cells were calculated on each group; (F) The IFN-β expression following by OMVs (0.5 ug/ml) stimulation for 2 h in normal or acidic (pH = 6.3) cell cultures, the peritoneal macrophages of mice were pretreated with DMSO or BX795(1uM) for 1 h; (G) Western blot analysis of the proteins in innate immune signalling pathway isolated from peritoneal macrophages of mice, which were pretreated with DMSO or BX795(1uM) for 1 h following by OMVs (0.5ug/ml) stimulation for different time points in normal or acidic (pH = 6.3) cell cultures; ***: *p* < 0.001.

Given that purified PA_OMVs could induce IFN-β expression, we next asked whether OMVs played an important role in the process of the increased IFN-β production followed by *P. aeruginosa* infection that promoted by acidic environment. We set acidic bacterial culture by adding substances such as lactic acid, hydrochloric or acetic acid. Results showed that the amounts of OMVs released by *P. aeruginosa* in acidic environment was significantly higher than that in normal environment (Figure 5(A), Figure S3A, 3B). Subsequently, we stimulated mice peritoneal macrophages with the same amounts of purified PA_OMVs in different culture environments to detect IFN-β mRNA expression and the activation of key proteins on the signalling pathway that led to IFN-β production. Results showed that compared with the normal culture, acidic environment significantly promoted IFN-β expression after PA_OMVs stimulation, while alkalic environment inhibited it (Figure 5(B)). Moreover, acidic environment significantly promoted the phosphorylation of IRF3 and IFN-β production after OMVs stimulation, while alkalic environment inhibited it significantly (Figure 5(C,D)). To corroborate these findings, we explored the difference in IRF3 activation, that was, to clarify the nuclear translocation of IRF3 under different cultures by confocal images. Following by the stimulation of PA_OMVs, the proportion of IRF3 translocated into the nucleus was significantly increased in acidic environment compared with that in normal or alkalic environment, while the acidic environment alone did not promote IRF3 entry (Figure 5(E)). Besides, IRF3 inhibition could significantly inhibit the enhanced IRF3 phosphorylation and the increased IFN-β mRNA expression promoted by acidic environment (Figure 5(F,G)).

### Targeted knockout of IRF3 extenuated *P. aeruginosa* pulmonary infection that exacerbated by acidification

**Figure 6.**
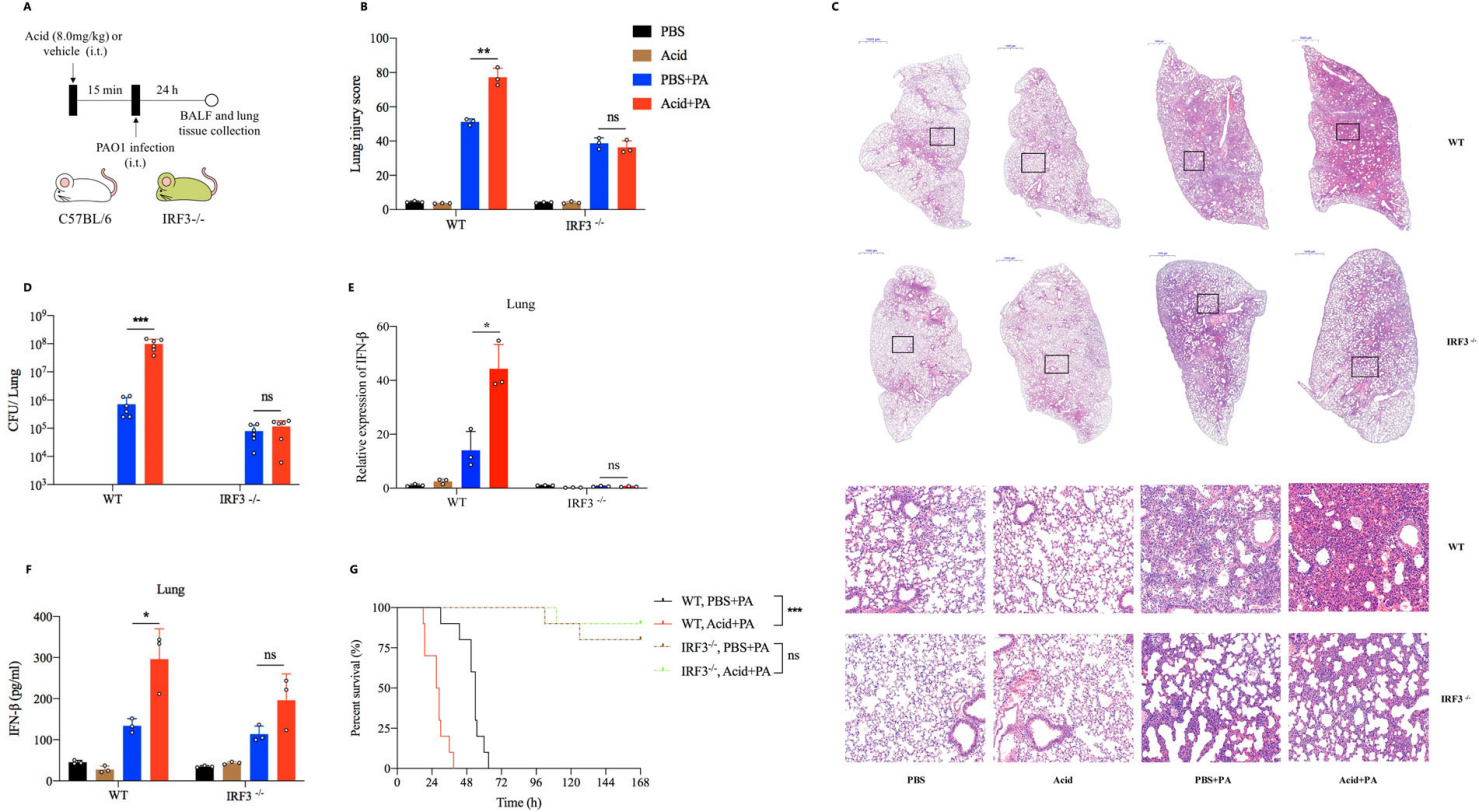
Targeted knockout of IRF3 reversed lung damage from *P. aeruginosa* infection that exacerbated by acidification. (A) Experiment and analysis scheme for PAO1 infection in WT and IRF3^−/−^ mice after acid pretreatment. IRF3 knockout mice with C57BL/6 background and age-matched WT mice were intratracheally infected with PAO1(2*10^6 cfu in 25ul PBS, per mouse) or PBS for 24 h with or without lactic acid pretreatment (8.0 mg/kg), and monitored for (B) and (C) the lung pathological damage following by H&E staining and lung damage scores, (D) the bacterial load, (E) IFN-β gene expression, (F) IFN-β protein production and (G) the survival (*n* = 10 per group); *p < 0.05; **p < 0.01; ***p < 0.001.

To explore whether targeting IRF3 therapeutically might alleviate *P. aeruginosa* pulmonary infection exacerbated by acidic environment, we adopt BX795, an IRF3 inhibitor, in vivo (Figure S4A), and found that IRF3 inhibition could significantly reduce the increased bacterial load (Figure S4B) and IFN-β expression (Figure S4C) in lung tissue of mice after *P. aeruginosa* infection that aggravated by acid pretreatment. Furthermore, we proceeded to investigate the effect of intratracheally lactate acid pretreatment on *P. aeruginosa* pulmonary infection model in IRF3^−/−^ mice (Figure 6(A), Figure S4D). Interestingly, IRF3 knockout significantly alleviated the increased lung damage (Figure 6(B,C), Figure S4E), bacterial load (Figure 6(D)) and IFN-β production (Figure 6(E,F)) in lung tissue following by *P. aeruginosa* infection that aggravated by acid pretreatment. Besides, IRF3^−/−^ mice were resistant to the increased lethality followed by *P. aeruginosa* infection that pretreated with acid (Figure 6(G)).

## Discussion

**Figure 7.**
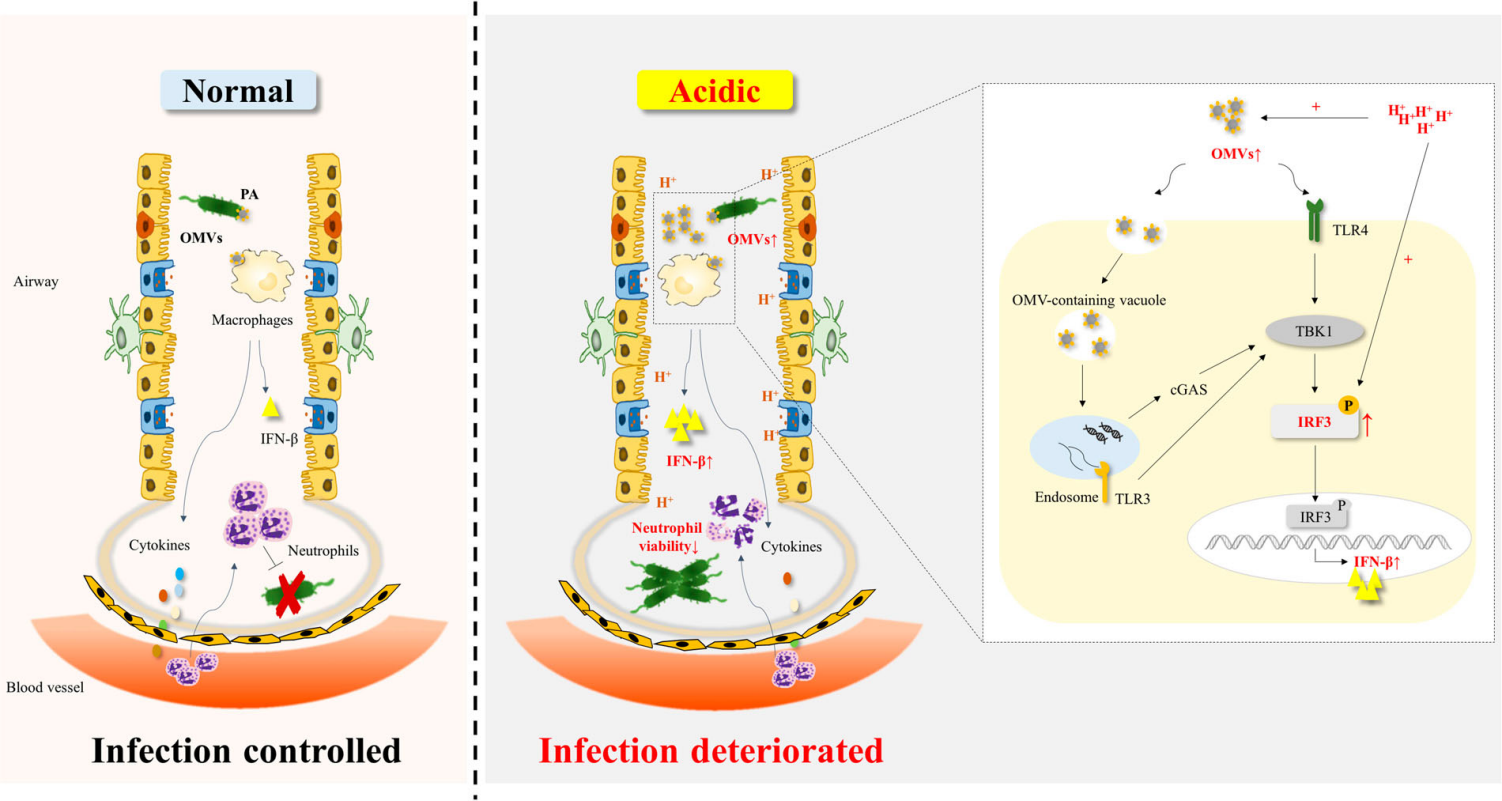
A model of acidic microenvironment mediated the amplification effect of type 1 interferonβ response following by *P. aeruginosa* infection. OMVs were involved in *P. aeruginosa* mediated the induction of IFN-β. Acidic microenvironment promoted the release of PA_OMVs and enhanced the phosphorylation of IRF3 induced by *P. aeruginosa* or PA_OMVs, leading to a significant increase in IFN-β production, which in turn led to the impaired host defense against *P. aeruginosa* pulmonary infection.

Data from this prospective cohort study suggested that EBC pH of patients with bronchiectasis was significantly lower than that of control subjects and correlated with clinical indicators including BSI and SGRQ scores. Acidic microenvironment could lead to the increased adhesion and invasion ability of *P. aeruginosa* to cells in vitro. Our study proposed a model in which acid microenvironment alarmed host by promoting the release of PA_OMVs and enhancing phosphorylation of IRF3 induced by *P. aeruginosa* or OMVs, thereby amplifying the type 1 interferon β response to impaire host defense against *P. aeruginosa* infection (Figure 7). Together, these findings indicated that targeting IRF3 displayed a protective role in *P. aeruginosa* pulmonary infection under acidic environment. The study not only clarified characteristics of airway microenvironment in bronchiectasis and the mechanism of disease deterioration caused by airway acidification, but also provided theoretical support for the need to pay attention to the pH of inhaled drugs in the future development of new drugs.

Oxidative stress was a major component of the mechanisms leading to the airway damage in bronchiectasis [32]. However, as an important oxidative stress marker in EBC, it was unclear whether EBC pH was associated with the disease characteristics of bronchiectasis. Our novelty findings suggested that airway acidification was strongly associated with the severity of stable bronchiectasis, patients with more severe disease had lower EBC pH. Marta PC, et al. had found that there was no significant relationship between EBC pH and clinical indicators including lung function tests and future acute exacerbations [33], which was somewhat different from our results. Only twenty-five stable bronchiectasis patients were included in their study, which may be difficult to show a potentially significant association due to the small sample size. Furthermore, the utility of ROC analysis in our study indicated that EBC pH had a good ability to discriminate severe bronchiectasis from mild or moderate patients, which suggested that measurement of EBC pH may be used as a simple noninvasive and auxiliary method to monitor disease severity in bronchiectasis.

*P. aeruginosa* was prone to drug resistance, which infection often led to high morbidity and mortality [34]. It was a classic gram-negative bacterium that could release OMVs spontaneously [20]. Previous studies had shown that purified OMVs could induce host inflammation responses through the activation of NOD, NF-kB signalling or caspase-11-dependent immune responses [22]. Interestingly, membrane vesicles secreted by *Escherichia coli* and *Staphylococcus aureus* could induce the expression of IFN-β, but whether the potent IFN-β induction of bacterial membrane vesicles had an impact on the pathogenesis of those infections remains unclear [35,36]. For the first time, we confirmed that PA_OMVs could activate a variety of PRRs, such as TLR4, cGAS and TLR3, to induce IFN-β production, which resulted in the deterioration of *P. aeruginosa* infection. It provided a theoretical basis for the treatment of refractory *P. aeruginosa* infection by targeting the inhibition of IFN-β production.

The importance of acid–base homeostasis in maintaining normal cellular responses and physiological integrity had long been recognized [37]. Acidic microenvironment played a role in suppressing immune function in cystic fibrosis and asthma [38,39]. In addition, studies had demonstrated that acidic microenvironment enhanced the release of pro-inflammatory cytokines such as IL-1β, IL-6 [40–43]. However, the effect of acidic microenvironment on the type 1 interferons response induced by bacterial infections was still unclear. Interestingly, our study revealed that acidic microenvironment could significantly enhance the activation of IRF3 followed by *P. aeruginosa* infection or OMVs stimulations, which in turn led to an increase in IFN-β production and promoted the colonization of *P. aeruginosa*, thus leading to the deterioration of infection. Mariappan et al. reported that the ability to adhere lung epithelial cells by *B.pseudomallei* was modulated by pH and glucose concentrations, without mentioning the underlying mechanism [44]. Our novel findings indicated that IFN-β blocking or inhibition could reduce the increased bacterial colonization and the aggravated host damage after *P. aeruginosa* infection that exacerbated by acidic microenvironment. This suggested the important role of IFN-β in aggravating *P. aeruginosa* infection under acidic microenvironment. Different physical factors, such as temperature, osmotic pressure, etc. may affect the release of exosomes from eukaryotic cells [45,46], while effects of environment pH on OMVs secretion were not well understood. Importantly, our study confirmed that weakly acidic microenvironment promoted the release of PA_OMVs, which ultimately further promoted the production of IFN-β after *P. aeruginosa* infection in acidic microenvironment. The detrimental role of type 1 interferons during *P. aeruginosa* lung infection had been revealed previously [47–50]. In this study, we focused on the impact of host airway microenvironment on bacterial infections and combined with the current hot topic of bacteria-virus co-infection, while verifying previous conclusions, we innovatively revealed that acidic microenvironment significantly promoted IFN-β production after *P. aeruginosa* infection and targeted knockout of IRF3 or IFNAR1 could reverse lung damage from *P. aeruginosa* infection that exacerbated by airway acidification. These provided new insights into the underlying mechanisms by which airway acidification aggravated the progression of some chronic airway inflammatory disease and provided a promising therapeutic target for the refractory *P. aeruginosa* infection.

Our study had several limitations. Firstly, induced sputum samples were not collected in our study, it was impossible to determine the correlation between EBC pH and the inflammatory cell infiltration in the airway of bronchiectasis patients. Secondly, considering that IRF3 was the dominant transcription factor during early type I interferons expression in most cells, we did not detect other interferon regulatory factors in this study, thus it cannot be ruled out the possibility that other IRFs may be also involved in the enhanced IFN-β response induced by *P. aeruginosa* infection under acidic environment. In addition, given that there was currently no perfect animal model of bronchiectasis disease, the acute *P. aeruginosa* infection mice model in this study was not aim to construct a disease model but to explore the potential impact of airway acidification on future acute *P. aeruginosa* pulmonary infection in bronchiectasis. Finally, it should be noted that EBC specimens were susceptible to saliva contamination, as well as patients’ eating habits and smoking status. Therefore, EBC-related studies needs to clarify patient sampling standards.

In conclusion, this study revealed that EBC pH could be developed as a good biomarker of the disease severity in stable bronchiectasis. Airway acidification promoted host type 1 interferons response through enhancing the activation of IRF3-IFN-β signalling pathway during *P. aeruginosa* infection. This shaded new light upon the question of detrimental role of airway acidification during *P. aeruginosa* infection. The study provided one more reference parameter for drug selection and new drug discovery for the treatment of chronic airway inflammatory disease that deteriorated by airway acidification.

## Supplementary Material

Supplemental Material

## Data Availability

The data generated in this study were available in the main text, the supplementary materials, the source data file were available after the article publication from jfxu@tongji.edu.cn upon reasonable request.
