# Supplementary material for: Airway acidification impaired host defense against Pseudomonas aeruginosa infection by promoting type 1 interferon β response
Source: Emerg Microbes Infect. 2022 Sep 14;11(1):2132–46. doi: 10.1080/22221751.2022.2110524 (PMC9487950; doi:10.1080/22221751.2022.2110524)
Supplement: Supplemental Material [file TEMI_A_2110524_SM9550.pdf]

Supplementary Materials for

Airway acidification impaired host defense against *Pseudomonas aeruginosa* infection by promoting type 1 interferon β response

Yang Liu[1,2#], Ying-Zhou Xie[1,2#], Yi-Han Shi[1,2#], Ling Yang[1,2#], Xiao-Yang Chen[3], Ling-Wei Wang[4], Jie-Ming Qu[5], Dong Weng[1,2], Xiao-Jian Wang[6], Hai-Peng Liu[7], Bao-Xue Ge[7], Jin-Fu Xu[1,2*]

* Corresponding author: E-mail: jfxu@tongji.edu.cn

SUPPLEMENTARY MATERIALS

Materials and Methods

Fig.S1. Acidic microenvironment promoted *P. aeruginosa* infection in vitro and aggravated its induced type 1 interferonβ response, related to figure2.

Fig.S2. Acidic microenvironment impaired host defense against *P. aeruginosa* infection by aggravating type 1 interferonβ response, related to figure3.

Fig.S3. Acidic microenvironment promoted PA_OMVs release, related to figure5.

Fig.S4. Targeted inhibition or knockout of IRF3 extenuated *P. aeruginosa* pulmonary infection that exacerbated by airway acidification, related to figure6.

Table S1. Gene-specific primers and probes

Materials and Methods

Clinical research participants-All enrolled patients were required to meet the following criteria：(1)Willing to sign the informed consent form and with good compliance; (2) Age>18 years, the diagnosis of idiopathic bronchiectasis needed to be referenced to the definition of "non-cystic fibrosis bronchiectasis guideline" published by British Thoracic Society in 2010, clinical symptoms of cough and expectoration, with or without intermittent hemoptysis, and chest CT showed bronchiectasis there; (3) All patients were clinically stable and had no evidence of infection or acute exacerbation for at least 4 weeks before the study. Acute exacerbation in patients with bronchiectasis was required to meet three or more of the following key symptoms for at least 48h: Cough; Sputum volume and/or consistency; Sputum purulence; Breathlessness and/or exercise tolerance; Fatigue and/or malaise; Haemoptysis, and a clinician determines that a change in bronchiectasis treatment was required[1]. Patients excluded from the acute exacerbation of bronchiectasis were defined as stable bronchiectasis. Subjects who meted any of the following criteria should be excluded from this study: (1) Patients with a history of other respiratory diseases (cystic fibrosis, allergic bronchopulmonary aspergillosis, asthma, 1-antitrypsin deficiency, pulmonary tuberculosis, COPD, lung cancer, interstitial lung disease), GERD and atopic diseases; (2) Receiving inhaled medications such as corticosteroids, antibiotics, or bronchodilators; (3) Participated in other interventional clinical trials within last 3 months; (4) History of smoking and alcohol abuse. Age and sex matched healthy controls had a negative history of allergy, normal lung function, no history of any lung disease (except for the history of pneumonia in the past and small pulmonary nodules) and without history of GERD.

EBC Collection and pH Measurement -Subjects were only allowed to drink water and no beverages were allowed during the 30 min before EBC collection. Firstly, placed the condenser at a higher level than the mouth to prevent saliva from entering the collection device; Secondly, confirmed that the equipment should apply one-way

suction valve to ensure that the patient did not inhale cold air through the condenser when inhaling; Then, all subjects were asked to inhale through their nose and exhale through their mouth at a normal breathing rhythm. EBC was collected with a disposable commercial available condenser (Rtube; Respiratory Research Inc., RRI). 10min was sufficient to obtain 1ml of sample and was well tolerated by subjects. All EBC samples should be frozen immediately after collection and stored at -80 ° C, with pH testing usually completed within 1 week. EBC samples were deaerated with argon at 350 ml/min until the pH reading was stable, which was measured using a pH microelectrode (Mettler Toledo). Calibration solutions with different pH values (pH=4, pH=7, pH=10) were used for accurate measurements.

Clinical data collection-Data on clinical characteristics, radiological features, EBC pH, blood gases indicators (PaO2, SaO2, PaCO2, pH, Lactic acid), lung function parameters (forced expiratory volume in 1s (FEV1), percentage of predicted FEV1 value (FEV1%), forced vital capacity (FVC), percentage of predicted FVC value (FVC%) and FEV1/FVC ratio), bacteriological results of sputum culture, modified Medical Research Council (mMRC) score, Bronchiectasis Severity Index (BSI) score and St George's Respiratory Questionnaire (SGRQ) score were uniformly recorded in this study. General conditions and EBC pH of all control subjects were recorded as well.

Regents used in this study- The following compounds were used: BX795 (IRF3 inhibitor, HY-10514), GSK8612 (TBK1 inhibitor, HY-111941), Resatorvid (TLR4/TRIF inhibitor, HY-11109) and Bafilomycin A1 (TLR3 inhibitor, HY-100558) were from MedChemExpress (Monmouth Junction, NJ, USA); Nocodazole (microtubule assembly inhibitor, CAS-31430-18-9) and Chlorpromazine (clathrin-mediated endocytosis inhibitor, CAS 50-53-3) were from TargetMol (USA); Ultra-LEAF™ Purified anti-mouse IFN-β Antibody (CAT 508108) and Ultra-LEAF™ Purified Armenian Hamster lgG Isotype Ctrl (CAT 400940) were from

BioLegend (USA). Recombinant Human IFN-β (CAT 300-02BC) was from PeproTech (USA), which had cross reactivity in mouse.

ELISA-IFN-β protein level was detected by ELISA kit (DY8324-05, R&D Systems) according to the manufacturer's instructions.

Confocal Microscopy-Peritoneal macrophages were incubated in the RPMI-1640 medium with different pH values (7.5, 6.3, 8.7), and stimulated with purified OMVs for 2h. Then cells were fixed with 4% formaldehyde for 30min at room temperature. After permeabilized with 1% Triton X-100 in PBS for 10min, the cells were blocked with 1% BSA in PBS for 1h at room temperature. Then, the cells were incubated with primary antibodies against rabbit anti-IRF3 (4302, Cell Signalling) overnight at 4 ℃. Washed cells with pre-cooled PBS for three time, and stained cells with Cy3-Labeled Goat Anti-Rabbit IgG (P0183, Beyotime) for 1h. Finally, stained with DAPI dihydrochloride for 15min and obtained confocal images with a Nikon confocal laser scanning microscope. At least 10 microscopic fields with more than 300 cells were calculated on each section.

Out membrane vesicles isolation and purification-The PAO1 strain was cultured in 200ml sterile Luria-Bertani (LB) broth for 13h (220rpm, 37°C) until the OD_{600nm} reached 1.0. The bacteria-free supernatant was collected by centrifugation at 10000 x g for 15 min at 4°C. This supernatant was further filtered through a 0.45 μm filter and OMVs were pelleted by ultracentrifugation at 400,000 x g for 1.5h at 4°C. After removing the supernatant, OMVs were resuspended in 100 μl sterile PBS and placed at 4°C for further analysis. Purified OMVs were subjected to agar plating to ensure lack of bacterial contamination and the total protein content of OMVs preparations was assessed by Pierce BCA protein assay kit (No.23227, Thermo Scientific) according to the manufacturer's instructions.

Transmission Electron Microscopy (TEM)-The morphologic observation of OMVs were performed in the same way as in a previous study[2] and were photographed under TEM (JEOL JEM-1230 (80KV)).

OMVs labelling and visualization of its uptake by mice peritoneal macrophage

To monitor whether PAO1_OMVs were taken up by macrophage, they were labelled with DiI (DiIC18(3)), a lipophilic carbocyanine fluorescent dye for membrane labelling (Synonyms: DiIC18(3))； HY-D0083, MCE), as described previously[3]. Briefly, the purified OMVs were resuspended with 1ml PBS in the presence of 1uM Dil and incubated at 37°C in water bath for 1h. Labelled OMVs were washed with PBS for three times to fully remove the unbound dye. After a final centrifugation step, the 20ug Dil-labeled OMVs were resuspended in 100ul PBS. OMVs internalization was assessed by confocal fluorescence microscopy. Peritoneal macrophages were stimulated with Dil-labeled OMVs (0.5ug/ml) for 2h in 15mm glass bottom cell culture dish (Nest, CAT 801002) at 37 °C in a 5% CO2 atmosphere. After the incubation, the cells were washed with PBS for three times to remove unbound or non-internalized OMVs, then fixed with 4% formaldehyde for 30min at room temperature. After permeabilized with 1% Triton X-100 (Sangon Biotech (shanghai) Co., Ltd, CAS 9002-93-1) in PBS for 10min, cells were blocked with 1% BSA (ST023, Beyotime) in PBS for 1h at room temperature. Then, the cells were incubated with primary antibodies against rabbit anti-ZO1 tight junction protein antibody [EPR19945-296] (ab221547) overnight at 4 ℃. Washed cells with pre-cooled PBS for three time, and stained cells with goat anti-rabbit IgG H&L (Alexa Fluor® 488) (ab150077) for 1h. Finally, washed cells with PBS for three time and stained with DAPI dihydrochloride (C1002, Beyotime) for 15min and obtained confocal images with a Nikon confocal laser scanning microscope (60 × oil immersion objective lens).

Quantitative Real-time RT-PCR-Total RNA was isolated from cells or lung tissue by using Triozl (Invitrogen, China), and the reverse transcription cDNA was synthesized by utilizing a Primescript RT reagent kit (Toyobo, Japan) according to the manufacturer's instructions. Real-time quantitative PCR measurement was performed in a volume of 10ul system by using SYBR Green reagent kit (Toyobo, Japan) and specific primers in ABI 7500 real-time PCR system (Applied Biosystems, USA)[4]. The gene-specific primers and probes were listed in Table S1.

Western Blot-Peritoneal macrophages or iBMDM were incubated in the RPMI-1640 medium with different pH values (7.5 or 6.3) and then stimulated with purified OMVs or PAO1 for different time. Subsequently, cells were lysed with 1* SDS loading buffer and boiled at 95°C for 20min to detect the expression of some proteins by immunoblot analysis. Samples were separated by electrophoresis through 10% or 12% polyacrylamide gels. Proteins was then transferred onto nitrocellulose blotting membrane (A29534166, GE Healthcare Life science) by electrophoresis and nonspecific binding was blocked with 5% BSA. Primary antibodies against rabbit anti-GAPDH (5174, Cell Signalling), against rabbit anti-STING (13647, Cell Signalling), against rabbit anti-TBK1(3504, Cell Signalling), against rabbit anti-phospho-TBK1(Ser172) (5483, Cell Signalling), against rabbit anti-IRF3(4302, Cell Signalling) and against rabbit anti-phospho-IRF3 (Ser396) (Cell Signalling) were used to incubate membranes overnight at 4℃. And then incubated with secondary antibodies for 1h. Proteins were visualized by using Amersham imager680 (AI680, GE, USA).

Quantification of bacterial load in lung tissue - The whole lung tissue was placed in 1ml sterile PBS and ground into homogenate with a tissue grinder (70HZ, 2min; SKSI, BiHeng Biotechnology lnc). 0.1ml of the homogenate was taken for gradient dilution, and then cultured it on LB agar at 37°C for 18h to confirm the bacterial load.

The remaining homogenate was centrifuged at 9000 rpm for 3 minutes, and the supernatant was frozen at 80°C for cytokine detection.

Lung Tissue Histology and Lung Injury Score-Left lungs were fixed with 4% paraformaldehyde, then embedded in paraffin and sectioned. Finally, completed hematoxylin-eosin(H&E) staining and observed under a light microscope. Lung histology scores were collected under blinded experimental conditions and were performed as in a previous study[5].

Flow cytometry-The following monoclonal antibodies were used for the flow cytometry cellular analyses: APC anti-mouse Ly6C (128016, Biolegend), Brilliant Violet 421™ anti-mouse Ly-6G (127627, Biolegend), ANTI-M CD170 1RNM44N PCP-EF710 (46-1702-82, Ebioscience), Ms CD11c PE-Cy7 HL3 (558079, BD Biosciences), Apotracker^{TM} Green (427402, Biolegend), 7-ADD Viability Staining Solution (420403, Biolegend). The whole lung was lavaged with 0.4 ml sterile PBS for a total of three times, immediately placed on ice, and then centrifuged at 400 g for 7 minutes (4°C). The supernatant of BALF was stored at -80°C for cytokine analysis. The cell pellet was re-suspended in PBS, and then count the total cell number in each sample after eradicating the red blood cells. Cells collected from BALF were incubated with appropriate dilutions of neutrophils, monocytes and macrophages antibody panel at 4°C for 30min. Then washed cells with PBS solution containing 1% FBS and 0.5% EDTA. Macrophages were identified as SiglecF^{high} CD11c^{+}Ly6G^{-}Ly6C^{-}; Neutrophils were identified as SiglecF$^{low/-}$ CD11c^{-}Ly6G^{+}Ly6C$^{low/-}$; Monocytes were identified as SiglecF$^{low/-}$ CD11c^{-}Ly6G^{-}Ly6C$^{low/high}$. Finally, the proportion of neutrophil, monocyte and macrophage cells were acquired with the Flow Cytometer (BD Accuri TM) and related data was analysed by FlowJo software 10.4 (FlowJo, LLC and Illumina, Inc).

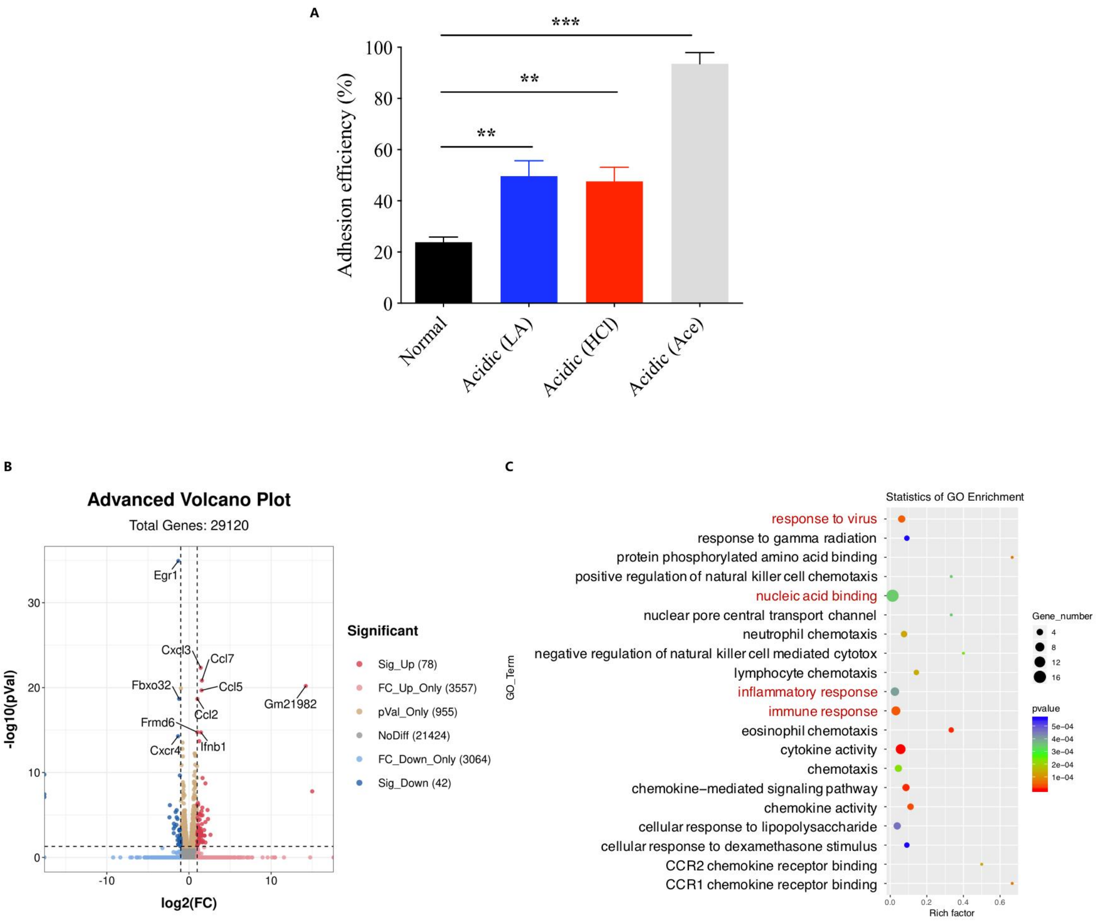

Fig. S1. Acidic microenvironment promoted *P. aeruginosa* infection in vitro and aggravated its induced type 1 interferonβ response, related to figure2

A) Comparisons of the adhesion ability of PAO1 to A549 cells in normal or acidic (pH=6.3) culture environments that adjusted by lactic acid (LA), hydrochloric (HCl) or acetic acid (Ace); The gene volcano map, GO enrichment scatterplot of mice peritoneal macrophages that stimulated by P. aeruginosa LPS in normal or acidic (pH=6.3) culture environments were shown in panel B) and C). **: p<0.01; ***: p<0.001.

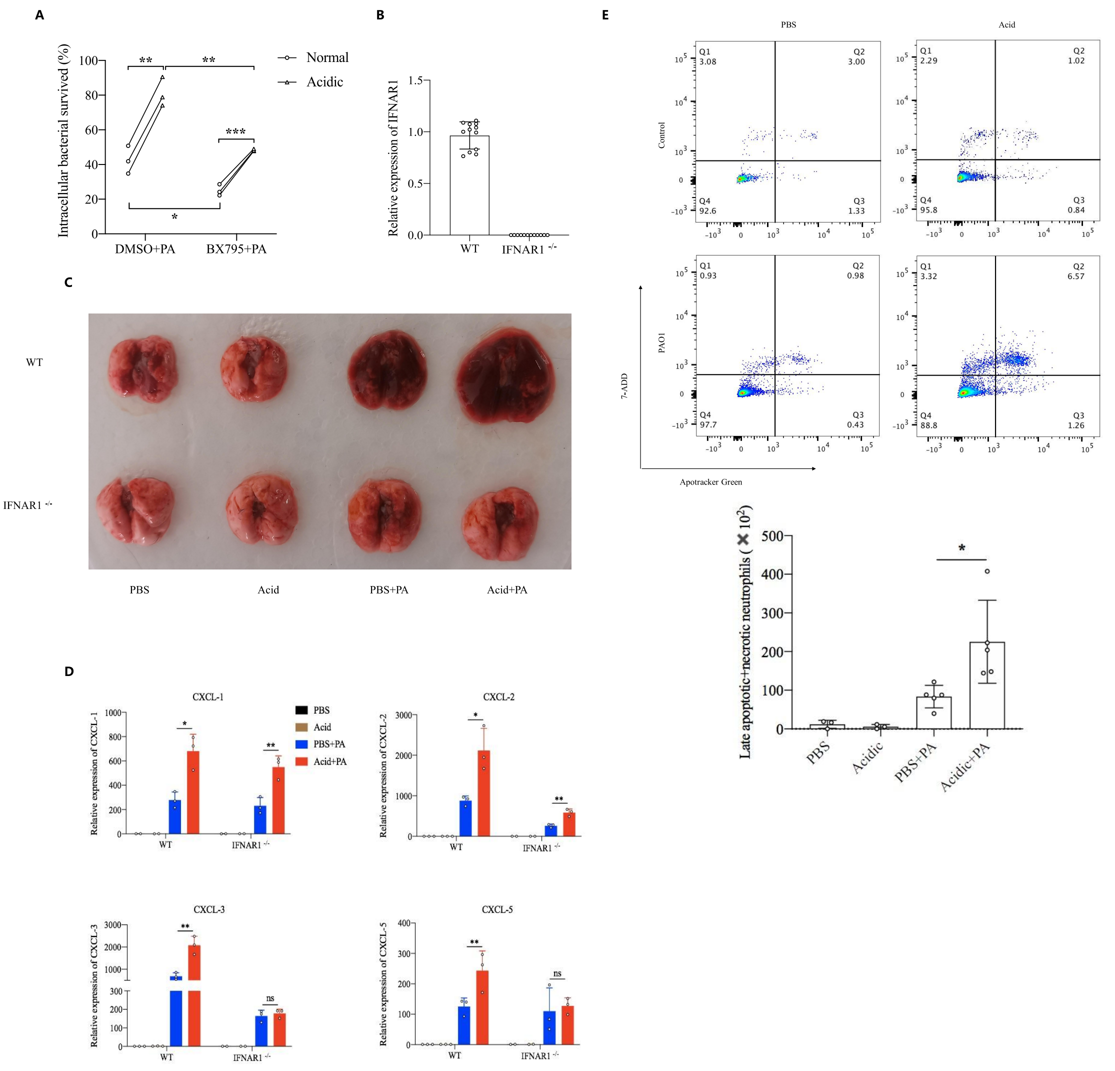

Fig. S2. Acidic microenvironment impaired host defense against *P. aeruginosa* infection by aggravating type 1 interferonβ response, related to figure3.

A) Comparisons of the intracellular bacterial survived rate of PAO1 in mice peritoneal macrophages in normal or acidic (pH=6.3) culture environments, cells were pretreated with DMSO or IRF3 inhibitor BX795(1uM) for 1h, then stimulated with PAO1(MOI=1) for 2h before lysis; B) mRNA expression of IFNAR1 in WT and IFNAR1$^{-/-}$ mice was assessed by real-time PCR; C) Representative lung images of WT and IFNAR1$^{-/-}$ mice after intratracheally infected with PAO1 (2*10^6 cfu in 25ul PBS, per mouse) or PBS for 24h with or without lactic acid pretreatment (8.0mg/kg); D) Comparisons of the CXCL-1, CXCL-2, CXCL-3 and CXCL-5 gene expression in WT or IFNAR1$^{-/-}$ mice lung tissue following by intratracheally infected with PAO1(2*10^6 cfu in 25ul PBS, per mouse) for 24h with or without lactic acid

pretreatment (8.0mg/kg); E) Quantification of the late apoptotic and necrotic neutrophils identified in the balf of C57BL/6 mice by flow cytometry. *: p<0.05; **: p<0.01; ***: p<0.001.

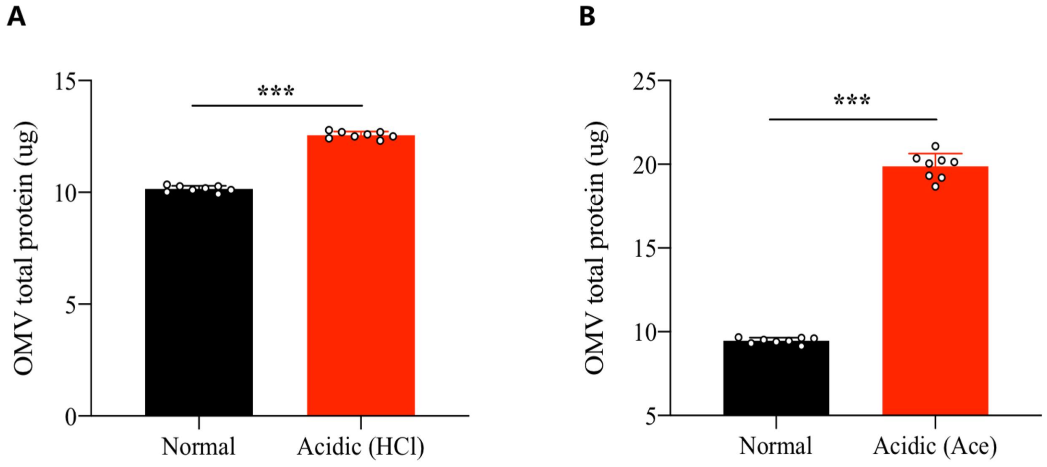

Fig. S3. Acidic microenvironment promoted PA_OMVs release, related to figure5

BCA protein quantitative analysis of OMVs total protein released by PAO1 in normal and acidic (pH=6.3) bacterial culture environments that adjusted by HCl or Ace were shown in panel A) and B). ***: p<0.001.

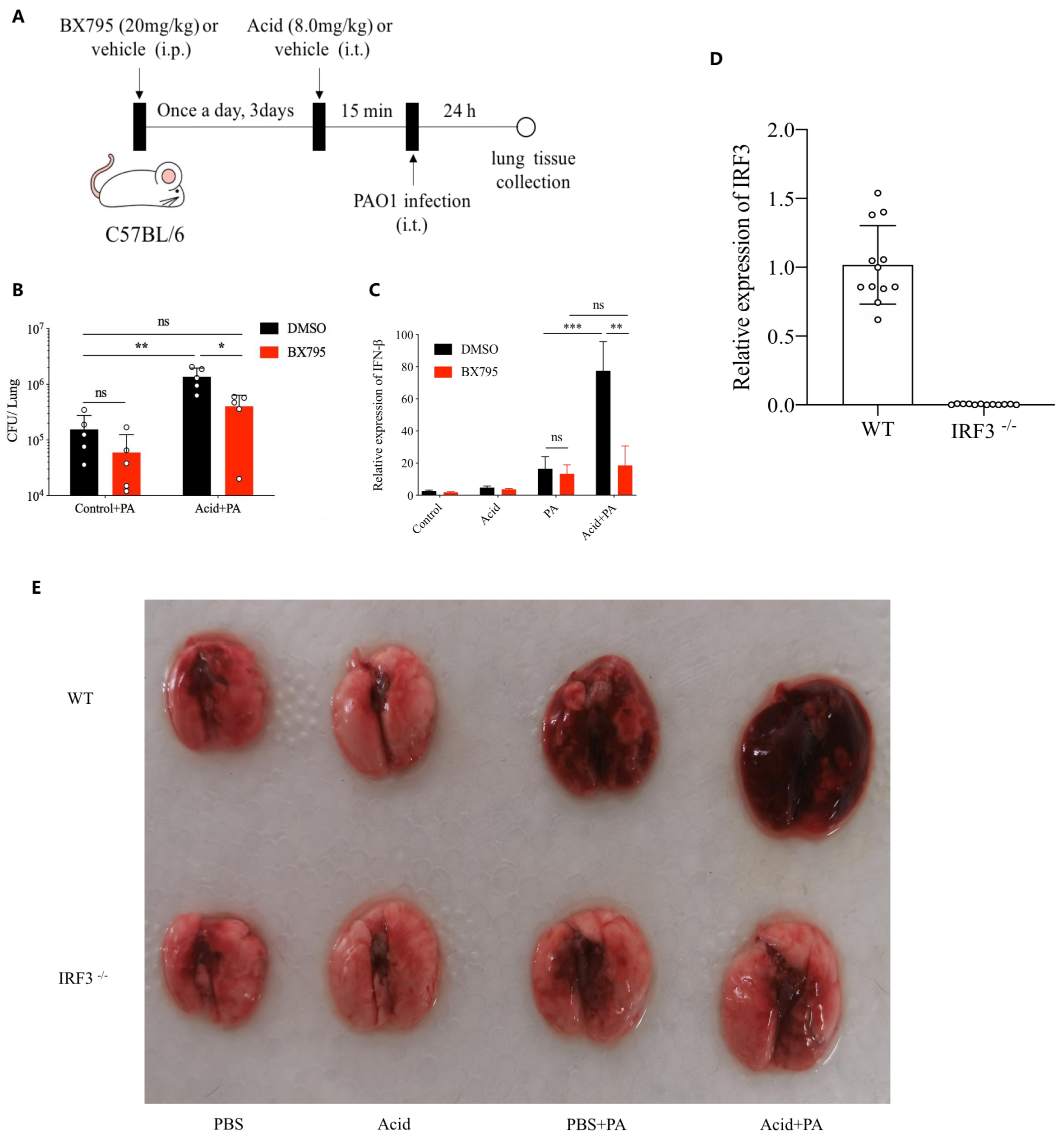

Fig. S4. Targeted inhibition or knockout of IRF3 extenuated *P. aeruginosa* pulmonary infection that exacerbated by airway acidification, related to figure6

A) Experiment and analysis scheme for the efficacy of BX795 (20mg/kg) pre-intraperitoneal administration for three days on *P. aeruginosa* pulmonary infection model with or without acid pretreatment. Six week old female C57BL/6 mice were pretreated with BX795 for three days and then intratracheally infected with PAO1(2*10^6 cfu in 25ul PBS, per mouse) or PBS for 24h with or without lactic acid (8.0mg/kg) pretreatment, and monitored for B) the bacterial load and C) IFN-β mRNA expression in lung tissue of mice; D) mRNA expression of IRF3 in WT and IRF3$^{-/-}$ mice was assessed by real-time PCR; E) Representative lung images of WT

and $IRF3^{-/-}$ mice after intratracheally infected with PAO1 (2*10^6 cfu in 25ul PBS, per mouse) or PBS for 24h with or without lactic acid pretreatment (8.0mg/kg).*: $p<0.05$;**: $p<0.01$; ***: $p<0.001$.

Table S1. Gene-specific primers and probes

Mouse primers	Forward 5' to 3'	Reverse 5' to 3'
IFN-β	CAGCTCCAAGAAAGGACGAAC	GGCAGTGTAACTCTTCTGCAT
CXCL-10	AACTGTACGCTGTACCTGCAT	GCATCGATTTTGCTCCCCTC
CCL-5	GCTGCTTTGCCTACCTCTCC	TCGAGTGACAAACACGACTGC
IRF-3	GAGAGCCGAACGAGGTTCAG	CTTCCAGGTTGACACGTCCG
IFNAR1	GACAACTACACCCTAAAGTGGAG	GCTCTGACACGAAACTGTGTTTT
IL-6	TGTGCAATGGCAATTCTGAT	GGTACTCCAGAAGACCAGAGGA
TNF-a	TGCCTATGTCTCAGCCTCTTC	GGTCTGGGCCATAGAACTGA
IL-10	GCTCCTAGAGCTGCGGACT	TGTTGTCCAGCTGGTCCTTT
pro-IL-1β	TCTTTGAAGTTGACGGACCC	TGAGTGATACTGCCTGCCTG
GAPDH	CCCACTAACATCAAATGGGG	CCTTCCACAATGCCAAAGTT

References

1. Hill AT, Haworth CS, Aliberti S, et al. Pulmonary exacerbation in adults with bronchiectasis: a consensus definition for clinical research. The European respiratory journal. 2017 Jun;49(6).
2. Cheng H, Fang H, Xu RD, et al. Development of a rinsing separation method for exosome isolation and comparison to conventional methods. Eur Rev Med Pharmacol Sci. 2019 Jun;23(12):5074-5083.
3. Hu R, Lin H, Li J, et al. Probiotic *Escherichia coli* Nissle 1917-derived outer membrane vesicles enhance immunomodulation and antimicrobial activity in RAW264.7 macrophages. BMC microbiology. 2020 Aug 27;20(1):268.
4. Huang HR, Li F, Han H, et al. Dectin-3 Recognizes Glucuronoxylomannan of *Cryptococcus neoformans* Serotype AD and *Cryptococcus gattii* Serotype B to Initiate Host Defense Against Cryptococcosis. Frontiers in immunology. 2018;9:1781.
5. Matute-Bello G, Downey G, Moore BB, et al. An official American Thoracic

Society workshop report: features and measurements of experimental acute lung injury in animals. American journal of respiratory cell and molecular biology. 2011 May;44(5):725-38.
